# Graphene-based nanomaterials for peripheral nerve regeneration

**DOI:** 10.3389/fbioe.2023.1306184

**Published:** 2023-12-18

**Authors:** Domenica Convertino, Maria Letizia Trincavelli, Chiara Giacomelli, Laura Marchetti, Camilla Coletti

**Affiliations:** ^1^ Center for Nanotechnology Innovation @NEST, Istituto Italiano di Tecnologia, Pisa, Italy; ^2^ Department of Pharmacy, University of Pisa, Pisa, Italy

**Keywords:** graphene-based materials, CVD graphene, graphene-based neural interfaces, peripheral nerve regeneration, nerve conduits

## Abstract

Emerging nanotechnologies offer numerous opportunities in the field of regenerative medicine and have been widely explored to design novel scaffolds for the regeneration and stimulation of nerve tissue. In this review, we focus on peripheral nerve regeneration. First, we introduce the biomedical problem and the present status of nerve conduits that can be used to guide, fasten and enhance regeneration. Then, we thoroughly discuss graphene as an emerging candidate in nerve tissue engineering, in light of its chemical, tribological and electrical properties. We introduce the graphene forms commonly used as neural interfaces, briefly review their applications, and discuss their potential toxicity. We then focus on the adoption of graphene in peripheral nervous system applications, a research field that has gained in the last years ever-increasing attention. We discuss the potential integration of graphene in guidance conduits, and critically review graphene interaction not only with peripheral neurons, but also with non-neural cells involved in nerve regeneration; indeed, the latter have recently emerged as central players in modulating the immune and inflammatory response and accelerating the growth of new tissue.

## 1 Peripheral nerve injuries and repair

Peripheral nerve injury is a global clinical issue, significantly impacting the quality of life for patients and implying a substantial socioeconomic impact (Raza et al., 2020; Felder and Ducic, 2022). When peripheral nerves undergo a traumatic injury, a sequence of pathophysiological events occurs at the site of the nerve injury, where the axons undergo Wallerian degeneration and the remaining Schwann cells (SCs) create a favorable environment for nerve regrowth towards the target organ, by forming bands of Bungner and releasing neurotrophic factors and extracellular matrix molecules (Faroni et al., 2015). In recent years, increasing attention has been devoted to the possibility to repair and regenerate nerve tissues by adopting targeted biomedical nanotechnology and tissue engineering approaches (Silva, 2006; Li et al., 2011; Ding et al., 2015; dos Santos et al., 2021). In fact, although peripheral nerves can spontaneously heal after traumatic injuries, poor regeneration outcomes are observed when surgical end-to-end nerve sutures are needed (i.e., in the presence of nerve segment loss) due to tension at the nerve repair site. To overcome this problem, a surgical approach employing grafts (i.e., small portions of a nerve tissue used to fill the gap of the nerve stumps) and tissue engineering nerve conduits (artificial structures used to bridge the nerve defects) is preferred (Faroni et al., 2015). The nerve conduits connect the nerve stumps and provide physical guidance for the axons, guaranteeing the correct connections of sensory and motor fibers of the distal and proximal stumps and the fast reinnervation of the motor end plates of distal target organs, to minimize the muscle fiber atrophy (Zhang et al., 2019).

Traditional direct suturing involves fascicular or epineural repairs. However, if the distance between nerve stumps exceeds 5 mm, grafting or conduits are utilized. The autologous grafts (i.e., autogenous donor nerves harvested from other parts of the patient’s body) are the gold standard due to their nontoxicity, non-immunogenic effects and good biocompatibility, but together with the allografts (i.e., nerve grafts from a donor) show limitations in terms of functional recovery (Faroni et al., 2015). Donor site morbidity, size mismatch between the injured nerve and the available donor nerves and significant healing times are some of the disadvantages of autografts, while tissue rejection and disease transmission are often related to allografts (Rebowe et al., 2018).

**FIGURE 1:**
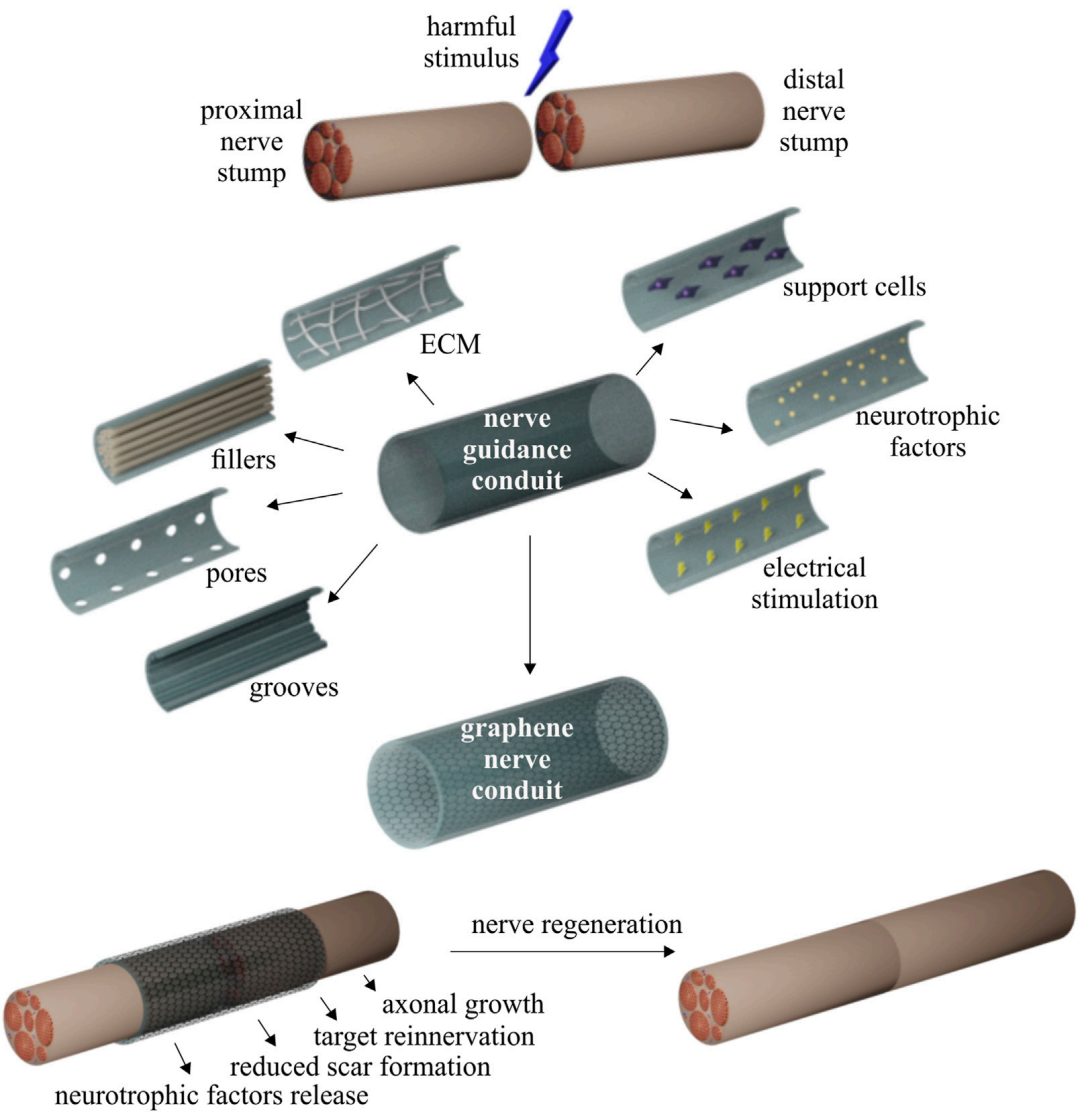
Peripheral nerve regeneration: experimental strategies and advantages of nerve guidance conduits.

To overcome these problems and satisfy the demand for high-performance nerve conduits, new promising alternatives have been proposed to heal the damaged nerve, including the development of biocompatible tissue engineered nerve conduits that mimic the structure of an autograft and provide enough support and mechanical strength while being flexible (Sarker et al., 2018) (Figure 1). The use of these nerve guidance conduits improves nerve regeneration by guiding an ordered axon outgrowth and reducing scar formation, allowing for nutrient and waste exchange through a porous structure (Faroni et al., 2015; Sarker et al., 2018). Ideally, the conduits should guide axonal growth towards the severed distal nerve by reducing axonal dispersion and off-target reinnervation and improving the neural biomechanical microenvironment following nerve injury. This could be achieved by including topographic and biophysical cues sensed by the cells via the cellular mechanotransduction (Kong et al., 2022). The incorporation of neurotrophic factors and support cells, such as SCs, stem cells and macrophages, is commonly used as neuroprotective therapy (Li et al., 2022b; Kim et al., 2022), while guiding cues, including intraluminal multi-channels, grooves in the inner wall and extracellular matrix-like structures are known to promote cell polarization and neurite/axon outgrowth (Chighizola et al., 2019; Scaccini et al., 2021). In addition, electrical stimulation (ES) is another method for accelerating the regeneration of injured peripheral nerves and enhancing their functional recovery, even in the presence of large nerve gaps (Huang et al., 2012).

Current clinically approved nerve guides are primarily made from synthetic or natural materials, including poly-glycolic acid (PGA), polycaprolactone (PCL), polyvinyl alcohol (PVA), type I collagen, chitosan and porcine small intestine submucosa (Rebowe et al., 2018; Fornasari et al., 2020). Among the materials that have been proposed for conduit production, silicon lacks long-term stability and biodegradability, while stimulating excessive scar tissue formation and requiring a secondary procedure for removal (Ciardelli and Chiono, 2006). An ideal conduit should be easily available and implantable and it should satisfy some of these requirements: be biocompatible; without inducing immunogenic reactions in the host tissue: be porous for nutrient and oxygen diffusion; be biodegradable to avoid a second surgery; be flexible and mechanically stable to support nerve regeneration without long-term compression; and be transparent to help the surgeons in prosthesis positioning by evaluating the alignment of the nerve stumps and to detect regenerated tissue in preliminary assessments without the need to cut open the conduit (Rebowe et al., 2018; Stocco et al., 2023). However, no materials can satisfy all these characteristics together: usually natural polymers, such as collagen and chitosan, show excellent biocompatibility, low immunogenicity and high bio-absorbability and sustainability, but lack adequate mechanical and electrical properties, while synthetic polymers are better in terms of versatility, mechanical properties and structural stability, but they have poor biodegradability and there are still concerns regarding toxic residual monomers from incomplete polymerization (Pinho et al., 2016; Boni et al., 2018). The synthetic polymers family includes also conductive polymers, such as polypyrrole (PPy), polyaniline (PANI) and polyethylenedioxythiophene (PEDOT), widely used to fabricate nerve conduits capable of influencing cell proliferation and axonal extension by appropriate ES (Guo and Ma, 2018).

Knowing that neurons are electrically excitable cells that transmit electrical signals, an electrically conductive material represents an ideal substrate for them. Indeed, it has been demonstrated that conductive materials can enhance the electric field produced by the cell membrane, influencing the bioelectric properties of the cells (Guo et al., 2016). ES can also improve and direct neurite outgrowth (Meng, 2014) and can promote axonal elongation (Fraczek-Szczypta, 2014). In recent years, electrically conductive materials have been proposed as alternative candidates for tissue engineering applications. Traditional conductive polymers, like Ppy, PEDOT and PANI exhibit favorable conductivity. However, they show often poor mechanical properties, low solubility in available solvents, and poor processability and biocompatibility (Kaur et al., 2015). Consequently, there has been a growing interest on conductive polymeric composites, realized by incorporating conductive fillers that form a conductive network though the composite while still maintaining the polymeric characteristics (Marsden et al., 2018). Among the fillers that have been utilized to increase the conductivity range of the biocompatible polymer networks, carbon-based materials such as carbon nanotubes (CNTs) and graphene, have attracted great attention, due to their good electrical conductivity, chemical stability and easy functionalization (Ding et al., 2015; Shin et al., 2016; Geetha Bai et al., 2018; Kumar et al., 2021; Qian et al., 2021; Hui et al., 2022; Bao et al., 2023).

## 2 Graphene for PNS regeneration

Graphene is a monolayer of sp^2^-hybridized carbon atoms arranged in a two-dimensional honeycomb lattice, first isolated from graphite in 2004 (Novoselov et al., 2004). It is a single layer of graphite that, due to the dimensional confinement, shows record electrical and thermal conductivity (Balandin, 2011), broadband light absorption (Bao and Loh, 2012), and exceptional mechanical properties (i.e., hosts extreme mechanical resistance and high flexibility) (Papageorgiou et al., 2017). The ensemble of these appealing properties opens up to the potential adoption of graphene for a large number of applications, which in the biomedical field span from biosensing to drug delivery and bio-imaging (Zhang et al., 2012; Reina et al., 2017). Furthermore, thanks to its carbon-based chemistry, graphene has been often presented as an excellent candidate material for neural interfacing devices (Kostarelos et al., 2017; Bramini et al., 2018). To date, significant focus has been paid to the perspective adoption of graphene-based materials (GBMs) in the central nervous system (CNS). The main applications include cell labeling and real-time live-cell monitoring, biomolecules delivering through the blood-brain barrier and highly sensitive electrodes that combine stimulation and recording and enable optoelectronic stimulation (Kuzum et al., 2014; Park et al., 2014; Bramini et al., 2018; Rauti et al., 2019). Since the integration of graphene with central neurons was observed to promote controlled elongation of neuronal processes, thereby facilitating neuronal regeneration (Li et al., 2011), GBMs have also been integrated in tissue engineering scaffolds (Ding et al., 2015; Bramini et al., 2018; Bai et al., 2019). These studies have paved the way to new research avenues, and while the CNS keeps being a central research topic, ever-increasing attention is being devoted to the adoption of graphene in the peripheral nervous system (PNS).

This review focuses on graphene and graphene-based neural interfaces and devotes particular attention to the potential use of graphene as a material for PNS regeneration, discussing how the complex system of cells involved in nerve regeneration could be affected by graphene. To date the overview of the adoption of different graphene and GBMs for this application is still incomplete. In fact, other reviews existing on the topic focus on graphene covalent-functionalized forms such as graphene oxide and reduced graphene oxide, or graphene flakes used in form of composites to design 2D or 3D scaffolds without including in the discussion other graphene forms (Grijalvo and Díaz, 2021; Aleemardani et al., 2022; Hui et al., 2022). As a matter of fact, the potentiality of planar highly crystalline pristine graphene in nerve regeneration is still little considered, although it has been reported in several works how the use of alternative graphene forms could improve the regeneration outcome (Convertino et al., 2018; Convertino et al., 2022). Herein, after a general introduction to nerve guide conduits for peripheral nerve regeneration, we elucidate graphene and GBMs synthetic methods and discuss the most common biomedical applications of these materials, their impact in central and peripheral neural interfaces, with a critical outlook on their potential cytotoxicity. We then discuss the existing studies on graphene and GBMs in nerve conduits, with a special focus on the potentiality of highly crystalline pristine planar graphene (i.e., epitaxial graphene grown on silicon carbide and graphene grown via chemical vapor deposition on copper or insulators). Finally, we address the interaction of graphene with different non-neuronal cell types involved in nerve tissue regeneration, a crucial point to understand and predict graphene performance *in vivo* upon implementation in nerve conduits.

## 3 Graphene and GBMs: production methods and main biomedical applications

Graphene and GBMs can be produced via different methods, resulting in different materials in terms of size, shape, number of layers, purity, lateral dimensions and chemical modification that can influence their application (Table 1) (Kostarelos and Novoselov, 2014; Backes et al., 2020). Recently, within the European Union’s GRAPHENE Flagship project, it has been proposed a classification system based on three physical-chemical properties of GBMs: the number of graphene layers, the average lateral dimensions, and the carbon-to-oxygen atomic ratio (Wick et al., 2014). Single-layer graphene is only one atom thick, in contrast, multilayer consists of a small number (2–10) of stacked graphene layers, named few-layer graphene if the layer number is between 2 and 5. Multiple stacking with a thickness up to 100 nm are referred to as nano-graphite, to distinguish them from the conventional thicker flakes of graphite powders (Bianco et al., 2013). Following the isolation of a single layer of graphene in 2004 via mechanical exfoliation, many efforts have been made to achieve large-scale synthesis of graphene (Li et al., 2021). Among the most common top-down production methods, liquid phase exfoliation (LPE) of graphite and reduction of exfoliated graphite oxide are typically used to produce dispersed graphene flakes. LPE of graphite, performed by ultrasonicating (or high-shear mixing) powdered graphite in solvents, yields graphene flakes with controlled thickness (from 1 to 100 layers), controlled dimension (lateral sizes from tens of nanometers to tens of microns), and good crystalline quality within a dispersing liquid (Backes et al., 2020; Tyurnina et al., 2021). While the resulting so-called graphene inks hold great potential for the realization of advanced composite materials and in energy conversion and storage, in the biomedical field the main applications include biosensing and intracellular delivery of small therapeutic molecules and drugs (Bonaccorso et al., 2016; Chen et al., 2023). For ultrahigh-sensitive biosensors, high-quality 2D crystals are usually required. However, for less demanding applications, such as electrochemical detection of analytes, LPE graphene has demonstrated good performances in detecting ascorbic acid, dopamine and uric acid (Qi et al., 2015; Banavath et al., 2022). Recently, LPE graphene was shown to enhance enzyme delivery to fibroblasts derived from patients with lysosomal storage disorders, demonstrating its potential as carriers for enzyme replacement therapy (Chen et al., 2023). Graphene oxide (GO), obtained by exfoliating oxidated graphite, is usually preferred for biomedical applications thanks to the easy functionalization of the oxygenated species introduced during the oxidation process (Smith et al., 2019). In addition, the resulting flakes are strongly hydrophilic and can be easily dispersed in water, with a broad potential in the biomedical sector. It can be easily integrated in a polymeric matrix, increasing surface hydrophilicity, therefore improving cell adhesion at the biomaterial surface (Pinto et al., 2013). However, in GO nanosheets, the large fraction of sp^3^ carbon covalently bonded to oxygen to form epoxy and hydroxyl groups degrades the electronic performances of the material that is an electrical insulator (Du et al., 2013). The GO properties can be improved by removing the oxygen functional groups via different reduction methods to produce reduced graphene oxide (rGO), however its properties are still poor if compared with pristine graphene due to the remaining oxygen-containing functional groups (Smith et al., 2019). But despite that, the presence of the functional groups in GO and rGO represents a great opportunity to tune their chemical and electrical properties (Li et al., 2021). Typical biomedical applications for GO and rGO include: 1) drug and therapeutic gene and cellular delivery, that take advantage of graphene chemical stability, the large surface area to increase the load rate and the easily functionalised surface chemistry; 2) phototherapy, that exploits GO photothermal effect to kill undesired cells and tumors; 3) biosensing, for which rGO is usually preferred with respect to GO for its higher conductivity; 4) bioimaging, that uses graphene as contrast agents in fluorescence, photoacoustic and magnetic resonance imaging; 5) sensing for RNA/DNA, glucose or disease biomarkers; and 6) antibacterial activity via oxidation and membrane stress (Bramini et al., 2018; Geetha Bai et al., 2018; Yan et al., 2019; Karki et al., 2020; Li et al., 2021; Cellot et al., 2022). Furthermore, GBMs such as LPE graphene, GO and rGO have been used in form of composites, foams, fibers and hydrogels to design tissue engineering 3D scaffolds with enhanced electrical and mechanical properties, to better mimic the *in vivo* environment (Bramini et al., 2018; Vlăsceanu et al., 2019; Savchenko et al., 2021; Lyu et al., 2022).

**TABLE 1:** Comparison between different synthesis methods of graphene and GBMs: advantages, disadvantages, and typical applications.

Synthesis methods	Description	Advantages	Disadvantages	Applications	Ref
**Liquid phase exfoliation (LPE) of graphite**	Graphite exfoliation in liquid environment followed by ultrasonication to separate the exfoliated flakes from the unexfoliated ones	Mass-production, low cost, tunability of chemical and electrical properties	Residual unexfoliated flakes to be removed, limited flake size	Fillers in composites; inkjet applications (electronic circuits, sensors, electrodes); filtration applications; energy applications	Fabbro et al. (2016), Tyurnina et al. (2021)
**Exfoliation of graphite oxide**	Obtained by graphite oxide exfoliation. Rich of oxygen-containing functional groups	Mass-production, low cost, hydrophilic, easily dispersed in water and polar organic solvents, tunability of chemical and electrical properties	Structurally defective, electrically insulating	Fillers in composites; coating for gas-barrier, corrosion resistance; antibacterial activity; sensors; drug/gene delivery carriers; photothermal therapy	Smith et al. (2019), Li et al. (2021)
**Reduction of graphene oxide**	Chemical or thermal reduction of the oxidation state of the oxygen functional groups to restore the sp^2^ structure	Electrically and thermally conductive	Low conductivity	Fillers in composites; energy storage; antibacterial activity; sensors; photothermal therapy	Smith et al. (2019), Li et al. (2021)
**Thermal decomposition of SiC**	Graphitization of SiC crystal surface following silicon atoms evaporation at high temperature. Mono to trilayer on the Si-face; multilayer on the C-face of SiC	Large-scale production, highly crystalline graphene, no need to transfer for specific electronic applications	High growth temperatures, large scale transfer challenging	Electronic devices; sensors; quantum Hall resistance standard	Riedl et al. (2007), Mishra et al. (2016), Convertino et al. (2022)
**Chemical vapor deposition on transition metals**	Thermal catalytic decomposition of a carbon precursor on metal. Monolayer growth on copper. Multilayer growth on nickel	Large-scale production, highly crystalline graphene, easy transfer on arbitrary substrates	Possible tears and breaks during transfer, metal and polymeric contaminants	Electronic and photonic devices; sensors; transparent electrodes; energy applications	Li et al. (2009), Miseikis et al. (2015), Saeed et al. (2020)
**Chemical vapor deposition on insulators**	Self-catalytic chemical vapor deposition or metal catalysis. Via a transition metal film deposited on sapphire	Large-scale production, highly crystalline graphene, easy transfer on arbitrary substrates, no metal contaminants (for metal-free approaches)	High growth temperatures, polycrystallinity	Electronic and photonic devices, sensors	Mishra et al. (2019), Li et al. (2022a)

Bottom-up synthesis of graphene, on the other end, allows one to obtain highly crystalline graphene on large (i.e., up to wafer) scale. To this end, thermal decomposition of silicon carbide (SiC) (Riedl et al., 2007), and chemical vapor deposition (CVD) of graphene on transition metals (Yu et al., 2008; Miseikis et al., 2015) as well as on insulators such as sapphire (Mishra et al., 2019; Li et al., 2022a) can be adopted. In the former case, graphene is formed after the sublimation of silicon atoms from the SiC crystal surface, whereas in the latter case, graphene is grown by depositing the precursor’s carbon atoms into a substrate.

Epitaxial graphene on SiC combines high crystalline quality, thickness homogeneity and an extreme cleanliness (Convertino et al., 2018). It can be used directly on the growth substrate that is known to be highly biocompatible, with no need to be transferred thus avoiding possible contaminants (Coletti et al., 2007; Oliveros et al., 2011; Convertino et al., 2022). Despite it has been demonstrated to be a valid interface for peripheral neuron integration, to date, epitaxial graphene on SiC remains an ideal platform for proof-of-concept investigations due to the high production costs and the non-transferability that impedes its integration with other substrates (Convertino et al., 2018).

Concerning transition metal substrates, nickel is usually preferred to grow multilayer graphene due to the high solubility of carbon in nickel (Yu et al., 2008), while the low solubility of carbon in copper allows the formation of a single layer graphene film (Li et al., 2009). To date, CVD monolayer graphene grown on copper has a quality comparable to the crystals obtained via mechanical exfoliation (Pezzini et al., 2020), and is largely adopted for applications that require high transparency and electrical conductivity including biosensing (Keisham et al., 2016; Singh et al., 2018), stem cell differentiation (Park et al., 2011) and neural electrodes (Kuzum et al., 2014; Park et al., 2014).

With respect to epitaxial graphene on SiC, the use of CVD graphene on copper holds potential for several applications, being the substrate cheaper and graphene transferreable to substrates of choice. Indeed, its flexibility and mechanical resistance as well as its excellent electrical conductivity have shown to yield positive effects on cell viability and axon elongation (Li et al., 2011; Lee et al., 2015; Convertino et al., 2020). To date, most of the studies that use planar graphene as a neural interface investigate its effect on CNS neurons. The use of CVD graphene, with or without a polymeric coating is actually preferred to realize 2D devices as planar transparent electrodes to stimulate and record neural activity. Indeed, recently graphene and CNTs have been successfully used to improve recording and ES of neurons (Keefer et al., 2008; Kuzum et al., 2014) and, surprisingly, neural microelectrode arrays (MEAs) fabricated using graphene obtained via CVD performed better than gold and indium tin oxide (ITO), in terms of signal-to-noise ratio (Rastegar et al., 2017). Park et al. developed a CVD graphene-based, carbon-layered electrode array device that was implanted in rodent brain for high-resolution neurophysiological recording. Thanks to graphene’s biocompatibility, transparency and flexibility, the device showed long-term *in vivo* stability and viability for optogenetic activation of focal cortical areas, electrophysiology and cortical imaging (Park et al., 2014). Kuzum and coauthors developed a transparent flexible neural electrode based on CVD graphene for simultaneous electrophysiological recording and optical imaging, with an improved signal-to-noise ratio and substantial reduction in electrical interference noise (Kuzum et al., 2014). The technique has been recently improved to integrate 2-photon microscopy, optogenetic, stimulation and cortical recordings in the same *in vivo* experiment (Thunemann et al., 2018), and it was used for multimodal monitoring of transplanted organoids for a comprehensive evaluation of the development, maturation and integration between the organoid and the host brain. Recently, CVD graphene has also been used to realize a solution-gated field-effect transistor that showed a performance similar to platinum black electrodes in recording visual and auditory responses in rats (Hébert et al., 2018), with long-term stability and biocompatibility in epicortical chronic implant (Garcia-Cortadella et al., 2021).

Park et al., 2011 exploited graphene as a transparent electrode, observing a good electrical coupling between graphene and neural stem cells (NSCs) for ES and confirming the neural activity of the differentiated cells. The resulting neural network functionality and the graphene effect on the maturation of the NSC’s electrophysiological state was also investigated by Guo et al., 2016. Graphene influenced both passive and active bioelectric membrane properties, hyperpolarizing the resting membrane potential and increasing the firing of action potentials during development, resulting in an accelerated maturation and enhanced neural performance (Guo et al., 2016).

The primary consideration when employing GBMs in biomedical applications is their biocompatibility. Indeed, the family of GBMs includes materials with widely variable properties that greatly influence the cell response (Wick et al., 2014). The layer number affects the elasticity and thus the adsorptive capacity of biomolecules that affect the cell interactions. The lateral size influences the cellular uptake, the blood-brain barrier crossing and the clearance from the body. The carbon-to-oxygen atomic ratio highly influence the structural properties and the surface chemistry, going from the highly hydrophobic pristine graphene to the GO that presents oxygen functionalities and hydrophilic regions (Wick et al., 2014). The following section investigates the effect of the production methods on the potential toxicity of GBMs to cell functions in neural interfaces.

## 4 Graphene and GBMs: potential toxicity in neural interfaces

### 4.1 Graphene nanosheet in neural interfaces

The preservation of neuronal health is key to many biomedical applications and crucial for the realization of scaffolds that enhance neuronal regeneration and functional recovery (Bramini et al., 2018; Lu et al., 2023). Indeed, the potential cytotoxicity of GBMs has been largely debated (Zhang et al., 2010; Liao et al., 2011; Fadeel et al., 2018). It has been demonstrated that GO induces functional alterations in primary astrocytes (Chiacchiaretta et al., 2018) and cortical neurons (Bramini et al., 2016). An impairment of excitatory transmission was found in primary neurons chronically exposed to graphene oxide flakes (Bramini et al., 2016; Rauti et al., 2016). Bramini et al. observed that, even without interfering with neuron viability and intrinsic excitability, GO exposure decreased the network electrical activity by creating an imbalance between synaptic excitation and inhibition (Bramini et al., 2016). Rauti et al., 2016 showed similar results on primary hippocampal neurons. They reported that high concentrations of GO affected synapse formation and function without altering cell survival. Cytotoxicity of graphene is often observed for dispersed graphene flakes: a shape and concentration-dependent cytotoxicity on PC12 cells was observed for graphene flakes (Zhang et al., 2010), just as GO had a dose-dependent cytotoxicity in human fibroblast cells and mice (Wang et al., 2011). Since the nanosheets are characterized by irregular protrusions and sharp edges, the cytotoxicity has been ascribed to their capacity to disrupt the cell membrane causing cell death (Bramini et al., 2018; Darbandi et al., 2018). Moreover, graphene aggregates could also induce oxidative stress, causing mitochondrial dysfunction, lipid peroxidation, or DNA damage (Zhao et al., 2014). However, there are still controversial findings on graphene nanosheet biocompatibility, probably due to the heterogeneity of the fabrication methods, that strongly influence the size, structure, charge, impurities and surface modification of the resulting graphene (Bramini et al., 2018; Fadeel et al., 2018). On the other hand, GO toxicity can be drastically reduced by functionalizing it with different coatings or reducing agents (Kuila et al., 2012; Yang et al., 2013). It is important to notice that the studies reported above explored the effect of graphene oxide nanosheets dispersed in the growth medium. On the contrary, when a flat surface coated with graphene produced by LPE was used, the synaptic formation and network activity in hippocampal neurons were not altered (Fabbro et al., 2016). This study reported that LPE graphene-coated glass coverslips retain unaltered neuronal behavior, supporting neuronal functional development without perturbing the neuronal network synaptic and electrophysiological properties (neuronal passive properties, spontaneous synaptic activity and short-term synaptic plasticity) (Fabbro et al., 2016). Moreover, uncoated thermally reduced graphene (TRG) was shown to favor multi-lineage differentiation of adult mouse olfactory bulb into neurons, astrocytes and oligodendrocytes. TRG supported the morphological differentiation of oligodendrocytes and the formation of functional synapses in neurons (Defterali et al., 2016). Overall, the above *in vitro* studies highlighted the negative effects of a chronic exposition to graphene sheets. It is worth noting that scaffolds incorporated with graphene may release flakes that could enter the bloodstream and be retained in various organs, becoming thus dangerous if such deposition occurs in large amounts. However long term *in vivo* studies that investigate the dependence of the effects on the size, dose and functionalization of the GO and rGO flakes are needed.

### 4.2 CVD graphene in neural interfaces

With respect to those GBMs, planar pristine graphene, a large-scale layer of sp^2^ hybridized carbon atoms, guarantees unique electrical and tribological properties, while presenting good biocompatibility. Indeed, even in the absence of a coating, CVD graphene was shown to be biocompatible, sustaining neuron survival and neurite outgrowth (Table 2). Bendali et al., 2013 examined the survival and neurite outgrowth of adult retinal neurons both on bare and polymer-coated graphene. They confirmed the graphene potential as a cytocompatible material for interfacing neurons with electronic devices, even though the presence of a polymeric coating seemed to help cell adhesion and spreading. Sahni et al., 2013 used bare graphene to culture cortical neurons and showed long, linear neurite growth and synapse-like structure formation. They also reported an increased adhesion of neurons on graphene, compared to bare plastic dishes, that was explained by van der Waals forces overcoming the hydrophobic forces of the plastic dish. In addition, CVD graphene was found to support the growth of primary hippocampal neurons, without affecting cell viability and morphology and accelerating neurite sprouting and outgrowth especially during the developmental phase (Li et al., 2011). More recently, Pampaloni et al., 2018 have disclosed that single layer graphene boosts neuronal activity, increasing the action potential frequency in hippocampal neurons by altering extracellular ion distribution at the material interface. They hypothesized that potassium ions are trapped at the carbon surface, leading to a local depletion at the neuronal membrane. Also, it has been shown, that neural affinity strongly depends on graphene quality. Veliev et al., 2016 showed that hippocampal neurons cultured on high quality bare graphene had an improved adhesion and outgrowth, with a neuritic architecture similar to conventional coated controls. On the other hand, the use of defective CVD graphene prevented cell attachment, demonstrating that the presence of carbon atoms alone does not guarantee the material cytocompatibility, but rather the material crystalline quality plays a crucial role. In contrast with these observations, Capasso and coworkers showed that highly defective graphene characterized by reduced electrical conductivity but comparable roughness and hydrophilicity with respect to highly crystalline graphene, did not affect viability, morphology, and electrical properties in hippocampal networks (Capasso et al., 2021).

**TABLE 2:** Impact of graphene and GBMs used as central and peripheral neural interfaces.

Type of graphene	Substrate	Sample	Graphene impact	Ref
**GO**	Dispersed in medium	Hippocampal neurons	Downregulation of neural signaling without affecting cell viability. Impairment of cell viability in larger flakes	Rauti et al. (2016), (2019)
**GO**	Dispersed in medium	Cortical neurons	Downregulation of excitatory transmission accompanied by a decreased density of excitatory synaptic contacts and upregulation of inhibitory transmission	Bramini et al. (2016)
**GO**	Dispersed in medium	Cortical astrocytes	Improved astrocyte-to-neuron communication	Chiacchiaretta et al. (2018)
**Functionalized GO**	Deposited on polyethyleneimine (PEI)-coated glass	Hippocampal neurons	Improved neurite outgrowth and branching in positively charged GO	Tu et al. (2014)
**G flakes**	Deposited on glass	Hippocampal neurons	No altered cell and synapse behavior	Fabbro et al. (2016)
**Epitaxial graphene on SiC**	On the growth substrate (SiC)	Dorsal root ganglion neurons	Survival on peptide coated and peptide-free graphene	Convertino et al. (2018)
**CVD monolayer on copper**	Transferred on tissue culture polystyrene	Hippocampal neurons	Neurite sprouting and outgrowth	Li et al. (2011)
**CVD monolayer on copper**	Transferred on sapphire	Retinal ganglion neurons	Survival on peptide-free graphene	Bendali et al. (2013)
**CVD monolayer on copper**	Transferred on glass	Hippocampal neurons	Neurite sprouting and outgrowth, improved dendritic network formation and synaptic activity	He et al. (2016), Pampaloni et al. (2018)
**CVD monolayer on copper**	Transferred on glass	Dorsal root ganglion neurons	Axon outgrowth through local stall of nerve growth factor signaling endosomes	Convertino et al. (2020)
**CVD monolayer on copper**	Transferred on glass	Hippocampal neurons	Improved adhesion and outgrowth. Repellent nature of poor-crystalline graphene	Veliev et al. (2016)
**CVD monolayer on copper**	Transferred on poly(ethylene terephthalate) substrates	Cortical neurons	No altered viability and excitability on graphene with different electrical properties	Capasso et al. (2021)

Despite the growing number of studies, the nature of the interaction between neurons and graphene, and the effect of different electrical, chemical and structural properties of graphene on neuronal activity, is still not completely clear (Bramini et al., 2016; Rauti et al., 2016; Chiacchiaretta et al., 2018; Pampaloni et al., 2018). Yet, the chemistry, morphology and tribological properties of planar graphene have shown to yield an overall positive effect on neural cell viability and proliferation, with no measurable cytotoxic effects.

## 5 Graphene and GBMs in peripheral nerve tissue regeneration

### 5.1 Graphene-based composites in peripheral nerve regeneration

The use of GBMs as peripheral neural interfaces to realize nerve conduits has been extensively explored by combining graphene and its derivatives with polymers, to realize composites, whose enhanced electrical conductivity is due to graphene sheets or CNTs inclusions (Deng et al., 2011; Heo et al., 2011; Golafshan et al., 2017; Liu et al., 2017), with promising results also *in vivo* (Jakus et al., 2015; Qian et al., 2018a; Qian et al., 2018b; Wang et al., 2019a; Wang et al., 2019b; Dong et al., 2020; Lu et al., 2023; Zhang et al., 2023).

**FIGURE 2:**
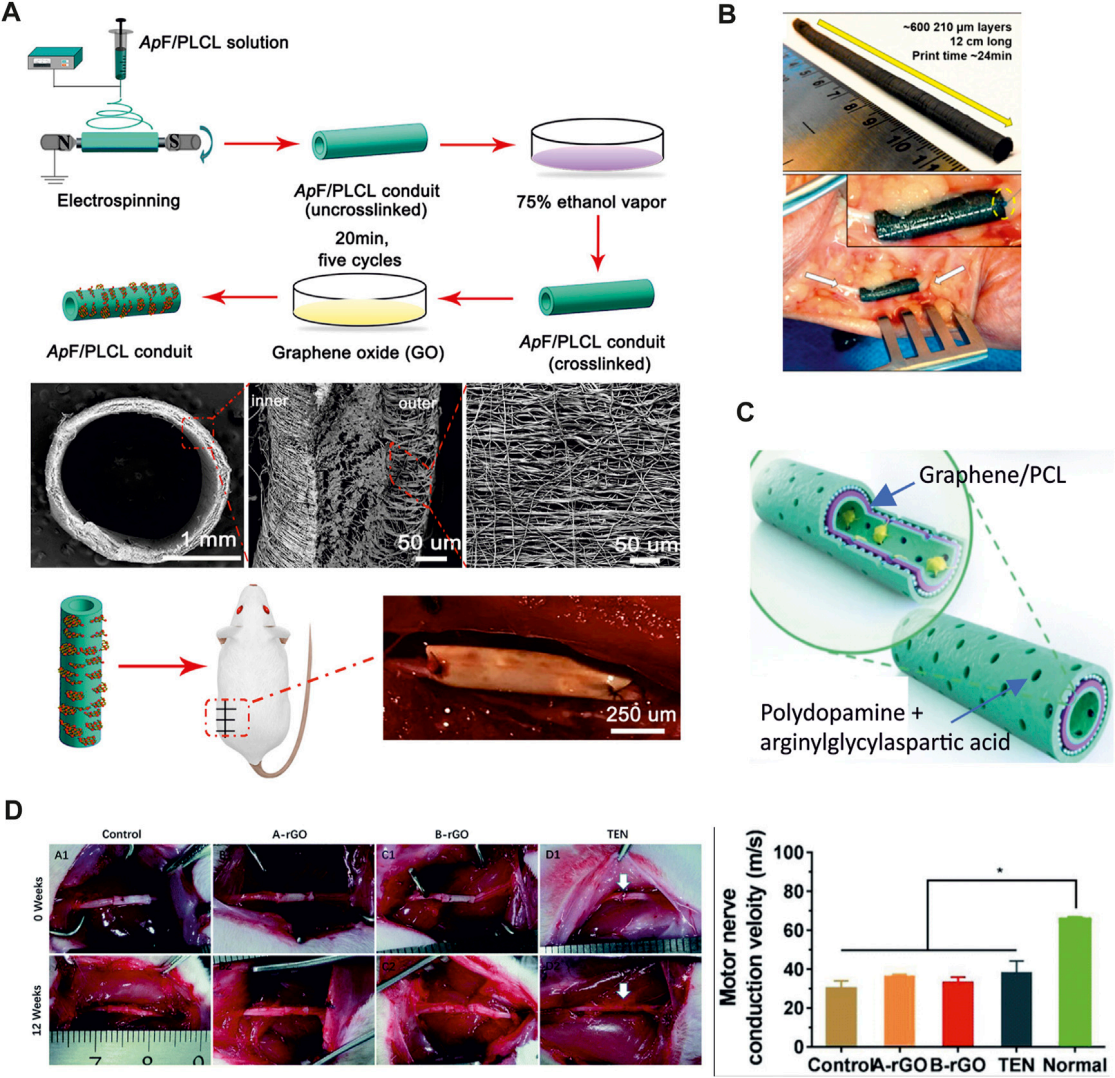
**(A)** Preparation of GO-ApF/PLCL nerve conduit and animal implantation. Schematic illustration of GO-ApF/PLCL nerve conduit preparation (top); characterization of GO-ApF/PLCL nerve conduit (center); optical images of nerve conduit at implantation (bottom) (Wang et al., 2019b). **(B)** 3D printed graphene nerve conduit wrapped around the ulnar nerve (Adapted from (Jakus et al., 2015). **(C)** Schematic illustration of the graphene loaded PCL conduit. The inner-most and outer-most green layers are PDA/RGD mixed layers. The purple layer is single-layered or multi-layered graphene and PCL mixed layer. The blue layer is a repetition of the graphene and PCL mixed layer (Adapted form (Qian et al., 2018b). **(D)** Surgical implantation of the GelMA/PCL nanofibers conduits (control, no rGO), rGO/GelMA/PCL at two different concentrations (A-rGO = 0.25 wt% rGO and B-rGO = 0.5 wt% rGO) and traditional surgical approach (TEN). The motor nerve conduction velocity of the B-rGO, A-rGO, Control, and TEN groups was comparable. Adapted from (Fang et al., 2020).

Many works examined the potential of GBMs in accelerating nerve tissue regeneration in the absence of an external ES, emulating the endogenous electric stimulation of the nerve tissue by contacting the conductive scaffold with the electrically active nerve tissue (Wang et al., 2019a). GO-coated nanofibrous scaffolds was demonstrated to create an ideal interface of SCs growth and successfully repaired a 10 mm sciatic nerve defect (Wang et al., 2019b) (Figure 2A). Silk fibroin-graphene hydrogels were shown to promote PC12 cell differentiation and neurite growth (Wang et al., 2019c). An enhanced PC12 cell adhesion and proliferation was also reported by Golafshan et al. in a hybrid graphene nanosheets-sodium alginate/polyvinyl alcohol fibrous scaffold (Golafshan et al., 2017). Gopinathan et al. illustrated the impact of electrically conducting carbon nano-fillers including carbon nano-fiber and nano-graphite in PCL-based porous scaffolds. All the nano-composite films supported PC12 attachment and differentiation, however, carbon nano-fiber-PCL based films were found to have better electrical conductivity and enhanced thermo-mechanical properties when compared to nano-graphite, and showed superior cytocompatibility (Gopinathan et al., 2016). The efficacy of graphite nanofilaments dispersed in an alginate hydrogel was reported in Homaeigohar and coworkers (Homaeigohar et al., 2019). Their hydrogel nanocomposite allowed to create local conductive zones, enabling intercellular signaling and provoking cells responses, with good biocompatibility also *in vivo*. Additional research supported the anti-inflammatory ability of 3D graphene foams, ascribed to their unique topographical features (Song et al., 2014). Indeed, 3D graphene evoked milder neuroinflammation in the microglia when compared to 2D graphene, suggesting that the topographical structures of the materials might affect the material/cell interactions and the inflammatory behaviors. Jakus et al., 2015 fabricated a 3D printable scaffold, consisting of graphene with a minority of polylactide-co-glycolide, that could be used as an electrically conducting scaffold for tissue regenerative engineering applications (Figure 2B). GO and CNTs were also embedded in positively charged hydrogel to realize a conductive nerve conduit to stimulate nerve cell differentiation (Liu et al., 2017). Recently, Qian and colleagues fabricated graphene nanoparticles and PCL scaffolds that improve *in vivo* axonal regeneration and nerve remyelination after physical nerve injury, with negligible toxicity and a successful sensory recovery even in a long nerve defect model (Qian et al., 2018b; Qian et al., 2021) (Figure 2C). They investigated the effect of both single and multi-layered graphene fillers. Although both graphene fillers induced successful axonal regrowth and remyelination after peripheral nerve injury, single-layered graphene conduit showed superior electrical and mechanical properties, knowing that multi-layered structure could compromise electric conductivity (Ding et al., 2015; Qian et al., 2018a), A small gap tubulization using a containing gelatin methacryloyl (GelMA), PCL and rGO was also reported to promote sensory and motor nerve regeneration and functional recovery of a transected sciatic nerve even, with a conduction function similar to the other control groups (GelMA/PCL without rGO and traditional epineurial neurorrhaphy) without applying ES (Fang et al., 2020) (Figure 2D). However, the application of a therapeutic ES was found to improve the repair and regeneration in peripheral nerve injury. Dong and coworkers incorporated graphene powders in a fibrous scaffold and applied exogenous ES demonstrating enhanced sciatic nerve regeneration and functional recovery (Dong et al., 2020). Moreover, they reported that daily stimulation exhibited significant therapeutic benefits in restoring motor and sensory function, nerve conduction function, targeted gastrocnemius muscle morphology, as well as promoting regeneration and remyelination of the injured nerve. These effects were notably superior to the brief ES and comparable to the gold standard autograft. In addition, a micropatterned polydopamine-decorated poly (l-lactide-co-caprolactone) (PLCL) conduit embedded with graphene powders was recently reported as a multifunctional guidance conduit that combines topographical features, surface coating and electric stimulation to promote neural regeneration, myelination, and the recovery of motor and sensory functions after peripheral nerve injury (Lu et al., 2023).

### 5.2 CVD and epitaxial graphene in peripheral nerve regeneration

**FIGURE 3:**
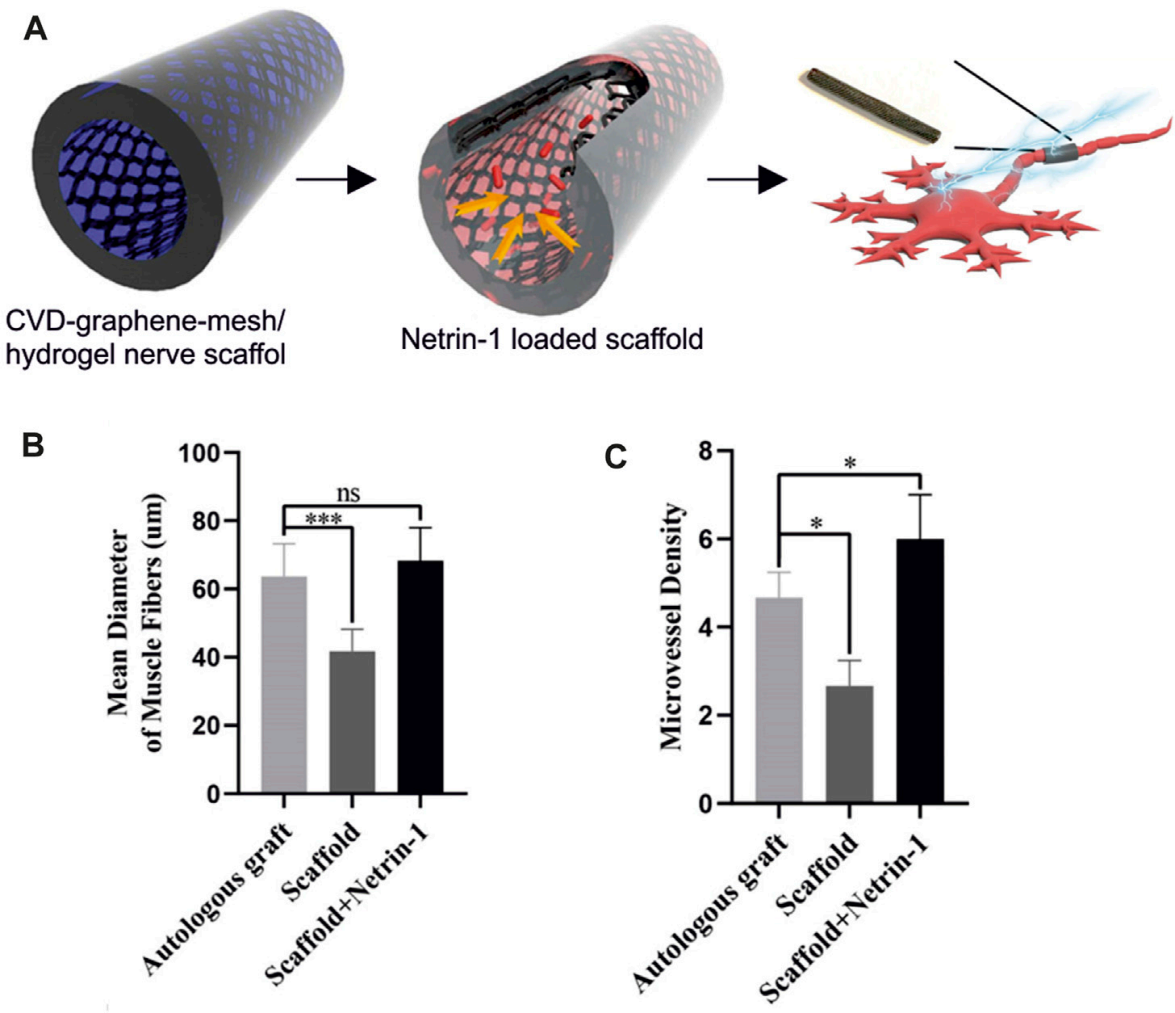
**(A)** Schematic illustration of netrin-1-loaded CVD-graphene-mesh/hydrogel nerve scaffold. **(B)** Mean diameter of muscle fibers shows no significant difference between the autologous graft group and the scaffold + netrin-1 group. **(C)** The scaffold + netrin-1 group shows a significantly higher angiogenesis of gastrocnemius muscles. Adapted from (Huang et al., 2021).

To date, the interaction between planar graphene and peripheral neural cells has scarcely been investigated, even if graphene positive effect on neurite outgrowth opens opportunities in neuroscience, neural engineering and regenerative medicine (Lee et al., 2012; Convertino et al., 2018; 2020). It should be stressed that the high electrical conductivity, flexibility and transparency required by these applications can be easily met by using CVD graphene. The interaction between planar graphene and PC12 cells, used as a model for peripheral neurons was reported for the first time by Lee and coworkers (Lee et al., 2012). They showed that CVD graphene coated with fetal bovine serum (FBS) enhanced neurite outgrowth and increased cell proliferation compared to bare glass coverslip. However, knowing that FBS is not a traditional coating for neural cells (Sun et al., 2012) and that it was used only for graphene and not for the glass control, little can be told about the impact of the graphene substrate *per se* on the results. The effect of pristine planar graphene with the traditional polymeric coating for neural cells on PC12 was then investigated by Convertino et al., 2018 using graphene grown by thermal decomposition on silicon carbide. In this work, it was shown that graphene stimulated a significant increase of neurite length in PC12 cells and that primary DRG neurons survived both on coated and uncoated graphene for more than 2 weeks. More recently DRG neurons were interfaced with CVD graphene transferred on glass and a significant increase of neurite length in the early days of culture was observed (Convertino et al., 2020). Also, the structural and dynamic information obtained when investigating the material effect on neuron physiology, revealed that graphene effect on axon elongation is mediated by the local stall of the nerve growth factor (NGF) signaling endosome in the early developmental stage (Convertino et al., 2020). The reduced excitability of DRG neurons was ascribed to the potassium ions adsorption on p-doped graphene, as previously suggested by Pampaloni et al., 2018. These studies provide relevant insights toward the understanding of graphene influence on neurite outgrowth and elongation, key information for using this material for neuroregeneration applications. Yet, flexibility remains a requirement for realizing nerve conduits that was not met in the reported works since the investigated graphene was grown or transferred on rigid substrates (i.e., glass and SiC). The integration of CVD graphene with flexible biocompatible substrates represents a central point for the development of high-quality graphene-based nerve conduits. In this direction, a 3D-graphene foam scaffold (CVD 3D-GF) grown via CVD on nickel was coupled with a biocompatible polymer to realize a conductive porous conduit (3D-GF/polymer) (Bahremandi Tolou et al., 2021). The study confirmed the efficacy of the conduit showing improved PC12 cell adhesion, proliferation and extension when compared to the same conduit without graphene. Similarly, Huang and coworkers combined a CVD graphene mesh with a hydrogel scaffold. It was loaded with the axonal guidance molecule netrin-1 and it was shown to promote angiogenesis and regeneration of the peripheral nerve and the restoration of the denervated muscle (Huang et al., 2021) (Figure 3). It is worth noting that, differently from conduits that use graphene flakes rather than CVD graphene, the use of CVD 3D-GF/polymer conduits is more promising due to their higher resistance to degradation, that avoids flakes release in the bloodstream. Also, interestingly, the percentage of graphene (2 wt%) in the polymeric matrix to achieve suitable mechanical and electrical properties was lower than the amount usually reported for graphene flakes, thus reducing the risk of toxic effects *in vivo* (Jakus et al., 2015; Bahremandi Tolou et al., 2021). In addition to 3D-graphene foam/polymer composites, CVD graphene was successfully transferred on a biocompatible and biodegradable copolymer, and used as a flexible conductive electrode for the ES of PC12 cells (Sherrell et al., 2014). Sherrel and coworkers drop-casted the biopolymers over graphene during fabrication, validating the ability to develop flexible biocompatible and conductive composites without using a graphene dispersion, but a continuous layer of high-quality CVD graphene. Additionally, integrating monolayer graphene with transparent polymers could also help in preserving the substrate transparency, a feature really appreciated by the surgeons being the conduit easier to handle and suture. Nevertheless, the development of this technique for engineering graphene nerve conduit requires a substrate-compatible transfer process for large-scale CVD graphene and is not compatible with the traditional manufacturing methods (e.g., dip coating, electrospinning, molding) and 3D printing (Han and Yin, 2022). Moreover, CVD graphene growth is more expensive than the common top-down synthesis, however many efforts have been made to develop a cost-effective synthesis method with controlled mass production and superior quality (Liu et al., 2022). Nevertheless, using CVD graphene in place of graphene flakes could have advantages in terms of regenerative results that might be worth to explore. The use of a continuous films could prevent agglomeration and neurotoxicity of nano-sized graphene (GO or rGO) while stimulate axon elongation and cell proliferation (Convertino et al., 2022). Moreover the electrically conductivity of rGO does not reach the one of pristine graphene, and rGO-based electrodes usually have low impedance and high charge-injection capacity that highly affect the recording and stimulation performances of the electrode (Bakhshaee Babaroud et al., 2022).

## 6 Graphene interaction with different non-neuronal cell types involved in nerve tissue regeneration

Following peripheral nerve injury several cell types besides neurons get involved. As soon as neurons get injured, SCs de-differentiate owing to their lost connection with axons, starting a vigorous proliferation and acquiring a precursor-like “repair” state, which accounts for the secretion of several pro-inflammatory factors such as interleukin (IL)-1, IL-2, IL-6, and tumor necrosis factor α (TNF-α) in the extracellular space (Balakrishnan et al., 2021). This promotes the recruitment of macrophages (Li et al., 2022b) to the injury site, which together with repair SCs are responsible for degrading and removing degenerated axons and myelin debris, a necessary step for axonal re-growth (Balakrishnan et al., 2021; Endo et al., 2022). The injury site is also rapidly invaded by fibroblasts (Parrinello et al., 2010) and other immune cells, including neutrophils (Kennedy and Deleo, 2009). Indeed, the innate arm of the immune system plays a crucial role in peripheral nerve regeneration (Kalinski et al., 2020).

Of note, other more cell types may be involved in the interaction with the injury site, when a cell therapy (typically, a stem cell therapy) approach is used either alone or in combination with the nerve conduit approach (Sullivan et al., 2016). The most thoroughly investigated cells for these applications are bone marrow mesenchymal stem cells, neural stem cells, adipose stem cells, skin derived precursor stem cells and, more recently, induced pluripotent stem cells (Yi et al., 2020). In this respect, understanding the interaction of these cell types with graphene is crucially important to unravel and predict its performance *in vivo* upon implementation in nerve conduits. For example, it was recently reported that GO and rGO-coated scaffolds, eventually coupled to ES, could positively affect SCs behavior by promoting the repair phenotype and by enhancing migration, proliferation, and myelination ability (Wang et al., 2019b; Fabbri et al., 2021; Aleemardani et al., 2022) as well as adipose mesenchymal stromal cells differentiation to SCs (Llewellyn et al., 2021).

GBMs were also shown to influence immune cells. Indeed, neutrophils were shown to recognize some carbon-based materials as pathogens, capturing and digesting GO and CNTs (Keshavan et al., 2019). Furthermore, GO and rGO were analyzed for their ability to influence macrophage response, and it was found that upon phagocytosis by the immune cells, they could decrease both oxidative stress and proinflammatory cytokine secretion, thus potentially boosting an appropriate immune response for tissue regeneration (Cicuéndez et al., 2021). Similar properties were ascribed to highly crystalline graphene particles (30–160 nm) derived by fragmenting CVD graphene grown on a nickel foam and phagocyted by human macrophages (Povo-Retana et al., 2021). The macrophage phagocytosis was confirmed also for pristine graphene flakes with an average size of 500 nm (Mcintyre et al., 2016). Concerning fibroblasts, monolayer CVD graphene was found to be non-toxic and to allow migration of murine subcutaneous connective tissue cells similar to controls (Lasocka et al., 2018). However, nano and micro sized GO were found to negatively impact embryonic fibroblast cell cycle and viability, underlying again the diverging performances that different graphene forms, with varying flake size, concentration and surface chemistry, can have (Liao et al., 2011; Hashemi et al., 2020). Finally, graphene was also studied for its ability to promote neural differentiation and affect the behavior of stem cells in the context of peripheral regeneration, as extensively reviewed elsewhere (Kenry et al., 2018; Yao et al., 2021; Hui et al., 2022). Conductive electroactive biomimetic scaffolds realized by combining GO with electrospun nanofibers were found to promote SC proliferation, migration and myelination, and to induce *in vivo* sciatic nerve repair with fiber regeneration similarly to the gold standard autograft (Wang et al., 2019b). Interestingly, graphene-based nerve conduits loaded with stem cell derived extracellular vesicles, i.e., biological nanoparticles displaying parental-cells-like pro-regenerative properties, has yielded promising results in an *in vivo* evaluation of peripheral nerve injury, while avoiding the issues of a cell therapy approach (Zhang et al., 2023). Overall, the interaction of GBMs with all the numerous cell types involved in peripheral nerve injury and regeneration remains largely unexplored, while it constitutes a crucial aspect to consider to avoid undesirable effects in designing graphene-based conduits. Further research is required in this direction to fully explore the implications of using graphene and GBMs-to achieve an optimal pro-regenerative performance.

## 7 Conclusion

In the field of neural regeneration, the evaluation of the cell’s response remains a relevant aspect. 3D scaffold should mimic the outer microenvironment, having physical, biological and biochemical features that favor cells proliferation, migration and differentiation. The majority of the FDA-approved conduits are made of bioresorbable polymeric hollow tube, that limits their repair efficiency to lesions less than 3 cm long (Meena et al., 2021). Despite the limitations of using hollow tubes, they still have a wide clinical acceptance when compared to most complex geometries. However, the use of innovative lumen wall designs, the addition of inner fillers and topological modifications, and the surface functionalization through conductive coatings have greatly improved the regenerative outcome. Among the conductive coatings that can be used to stimulate, direct and accelerate peripheral nerve regeneration, graphene and GBMs are bringing new perspectives thanks to their excellent conductivity and mechanical strength (Parker et al., 2021). To date, graphene oxide and graphene covalent-functionalized forms are preferred to realize three-dimensional scaffolds for *in vivo* nerve regeneration. They have been applied in polymeric composites, following the conduit topographical cues and allowing the scaffold loading with molecules, growth factors and stem cell, thus demonstrating a good integration with the current technologies (Jakus et al., 2015; Qian et al., 2018b; Qian et al., 2018a; Wang et al., 2019a; Wang et al., 2019b; Lu et al., 2023; Zhang et al., 2023). Yet, due to the possible cytotoxic effect of graphene nanosheets on nerve cells, further long-term investigations are needed (Fadeel et al., 2018). Planar graphene–such as graphene obtained via chemical vapor deposition and by thermal decomposition of SiC–has shown to have reduced cytotoxic effects (Fabbro et al., 2016; Veliev et al., 2016; Convertino et al., 2022). Indeed, CVD graphene has been successfully adopted for the realization of *in vivo* planar electrodes, which have been used for neural imaging and optogenetic applications (Kuzum et al., 2014; Park et al., 2014; Thunemann et al., 2018). Although conductive electrodes are the main field of application for planar graphene, the possibility to use CVD graphene to design implants should not be excluded. Indeed, CVD graphene has been reported to have a positive effect on axonal outgrowth of peripheral neurons, a result which suggests its possible use as an active conductive substrate for nerve regeneration (Convertino et al., 2020). The integration of CVD graphene with commercially available polymeric conduits adopted in neural regeneration might ultimately improve their electrical conductivity, while preserving the optimized biocompatibility and mechanical properties of the polymer. However, also for CVD graphene possible foreign-body reactions, long term degradation and bioresorption need to be carefully analyzed when considering *in vivo* applications. As discussed in this review, different graphene forms differently affect the neuronal and non-neuronal cells involved in the regeneration process (Kostarelos and Novoselov, 2014). For this reason, additional systematic research is needed in the coming years to truly unveil the effective potential of graphene and GBMs for the realization of novel nerve conduits meeting the diverse and complex requirements emerging at the nerve injured site.
